# Individualising drug therapy in intensive care

**DOI:** 10.1177/0310057X251404175

**Published:** 2026-01-23

**Authors:** Jacqueline A Hannam, Brian J Anderson

**Affiliations:** 1 Department of Pharmacology and Clinical Pharmacology, University of Auckland, New Zealand; 2 Department of Anaesthesiology, University of Auckland, New Zealand

Medical research often occurs in silos. This is very evident within the intensive care community where the double-blinded randomised trial is considered the gold standard for testing medical interventions because it gives the most reliable evidence about whether a treatment truly works. The double-blinded randomised trial may not be the best research methodology when it comes to studies involving pharmacology, particularly dose determination. Other types of methodology that use pharmacological principles have proven superior.^1^ Such research requires the involvement of pharmacologists skilled in the use of pharmacokinetic–pharmacodynamic (PKPD) modelling.

Safe medicine administration policies often revolve around the five rights (right patient, right medication, right dose, right time, right route), but right dose can be the tricky ‘right’. Dose depends on an understanding of pharmacokinetics (PK), pharmacodynamics (PD) and the adverse effect profile for the individual. Those patients cared for in the intensive care environment can prove to be very difficult to determine the right dose. In this issue of Anaesthesia and Intensive Care, Xu et al.^2^ describe the use of population PK modelling to understand cefazolin concentration changes in a critically ill patient with sepsis who was undergoing prolonged intermittent kidney replacement therapy. An understanding of PK in this patient allowed prediction of the cefazolin dose that was associated with therapeutic effect, in this case, the proportion of time (T) that the unbound drug concentration (*f*) remains over the causative organism’s minimum inhibitory concentration (MIC) (100% *f* T > MIC).

The heterogeneity of the intensive-care-unit population, concomitant drug use, routes of administration and their occasional extreme degrees of physiological derangement present PK, PD and drug safety challenges. Incorrect dose in patients with hepatic or renal dysfunction risk accumulation of both parent drug and metabolites. Underdosing of beta-lactam antibiotics has been described in those children with sepsis with increased creatinine clearance.^3^ Children present further challenges related to maturation and size. Even studies using population PKPD methodology to determine dose in intensive care patients can prove difficult to complete.^4^

Dose determination methodology has improved slowly. Some older methodology still exists. It remains common for aminoglycoside antibiotic dose to be determined using a single trough concentration measure, a best-guess method, rather than employing pharmacological principles. Some might look at peak as well as trough concentration to get an idea of half-life. Those who truly seek a degree of individualised treatment might determine clearance (CL) for antibiotics with a small volume of distribution (V). There is no single parameter that can, alone, describe the nonlinear relationship between concentration and effect.^5^ Even the somewhat more sophisticated parameter of CL, the primary parameter that determines infusion rate, is inadequate without understanding the interplay between PK and PD.

Xu et al.^2^ have used a population modelling approach in an individual with complex physiology. This is a good step toward individualised dosing, but it is only in one individual. It is difficult to extend this understanding to other patients in intensive care, given the complexity of the intensive care environment and often-large between-patient variability. In addition, we are left with many unanswered questions. The PK of the patient before dialysis and how this compares with others in intensive care wards remains unknown. The effect of this type of dialysis, or indeed, other dialysis systems, on clearance, remains poorly quantified. The authors explored both one compartment and two compartment models, but it remains uncertain which model would better predict dose. An approach that would benefit other patients in the intensive care environment would be to develop an understanding of PKPD at a population level rather than at an individual level.

The principles of concentration-guided dosing provide a rational basis for personalised dosing^6^ and it is these principles that guide our use of target-controlled infusions in anaesthesia and intensive care medicine. Infusion pumps are loaded with population PK parameter estimates (CL, V) that can be used to achieve a target concentration that was determined using PD parameter estimates (E_max_, EC_50_). Individualisation is sought using covariate information such as age and weight. These are the pharmacological principles used for propofol infusion in both anaesthesia and intensive care medicine.^7^ These principles can be similarly applied to antibiotic dosing.

This type of dosing review is commonly known as target concentration intervention. A target effect (e.g. Bispectral Index 50; 100% *f* T > MIC) is chosen, and that effect is associated with a target concentration. Pharmacokinetic information is then used to achieve the target concentration using a predicted dose. Dose individualisation can then be considered at a population level (e.g. all patients administered propofol) or at a more specific group level (e.g. obese elderly adults). A truer individual dosing system uses observations of patient response to inform about PK and PD in the individual to then tailor their dose.^8^ This is a step beyond therapeutic drug monitoring.^9^ It requires a greater understanding of CL and volume changes in critically ill patients, as well as changes during renal replacement therapy. However, once understood, clinicians can then be provided with individualised prescribing software such as Model Informed Precision Dosing and that would be a major step forward.^10^

This software, loaded with the necessary PK and PD parameters is already used in some patient groups (e.g. oncology). Although already used for some antibiotics (e.g. vancomycin) in the intensive care ward, it should be extended to intensive care patients maintained on support systems such as dialysis or extracorporeal circuits. The case report by Xu et al.^2^ makes use of samples obtained in a traditional therapeutic drug monitoring paradigm but extends these to apply a population modelling approach. It is limited in application to only a single individual patient, yet it reminds us that for complex PKPD questions, population modelling should be the new starting point. Australasia is recognised for quality intensive care management and research. The PKPD modelling skills in the Australasian community are strong.^11^ We need a stronger partnership between these two groups that somewhat exist in silos, currently. We need a marriage.
